# CeO_2_ Induced Ni-Ce Interaction Enables Efficient CO_2_ Methanation on Ni/Al_2_O_3_/SiC Structured Catalyst

**DOI:** 10.3390/ma19122612

**Published:** 2026-06-17

**Authors:** Jiyue Xu, Jiaxin Qian, Xiangli Liu, Fei Gao, Yiqing Zeng, Shule Zhang, Zhaoxiang Zhong

**Affiliations:** 1 School of Environmental Science and Engineering, Nanjing Tech University, Nanjing 211816, China; 2 State Key Laboratory of Materials-Oriented Chemical Engineering, National Engineering Research Center for Special Separation Membrane, Nanjing Tech University, Nanjing 211816, China; 3 School of Chemistry and Chemical Engineering, Nanjing University of Science and Technology, Nanjing 210094, China; 4 NJTECH University Suzhou Future Membrane Technology Innovation Center, Suzhou 215300, China

**Keywords:** CO_2_ methanation, Ni/Al_2_O_3_ catalyst, Structured catalyst, CeO_2_ modification

## Abstract

Structured catalysts are one of the most widely used catalysts for carbon dioxide (CO_2_) methanation, while the interactions between the loaded catalyst and structural supports significantly affect its catalytic activity and stability. Herein, CeO_2_ modification was employed to regulate the effect of the interaction between Al_2_O_3_ and SiC on the Ni/Al_2_O_3_/SiC structured catalyst for CO_2_ methanation. The results of characterization and catalytic performance tests reveal that CeO_2_ promotes the formation of Ni-Ce interaction, which induces the generation of abundant oxygen vacancies and weakens the Al-mediated constraint on Ni reducibility due to Ni-Al_2_O_3_ interaction, thereby significantly boosting both activity and stability. Therefore, the optimal Ni-16CeO_2_/Al_2_O_3_/SiC catalyst exhibits a much higher CO_2_ conversion at 300–400 °C and superior stability during an 80 h long-term test in comparison with the Ni/Al_2_O_3_/SiC catalyst. This work provides a feasible strategy for designing a high-performance structured catalyst for CO_2_ methanation.

## 1. Introduction

Carbon capture, utilization, and storage (CCUS) is widely recognized as a pivotal technology to achieve deep emission reductions and carbon neutrality. Converting captured CO_2_ into high-value chemicals or fuels is an important process in CCUS, which not only achieves carbon emission reduction but also enables carbon resource recycling [1]. Compared with other CO_2_ conversion processes (such as methanol production, hydrogen production, or liquid fuel production), CO_2_ methanation requires no costly energy storage equipment, which enables the integration of green electricity into existing gas networks, achieving ultra-high-capacity, cross-seasonal energy dispatch and negative carbon emissions [2,3]. Owing to the high C=O bond energy of 803 kJ·mol^−1^, efficient catalysts are required to lower energy consumption in CO_2_ methanation [4]. Nowadays, a series of catalysts (such as nickel-based [5], copper-based catalysts [6] and others [7]) have been developed to achieve highly efficient hydrogenation of carbon dioxide to methane.

Structured catalysts prepared by coating a catalyst on a structural support have emerged as a research focus due to their advantages of extremely low bed pressure drop, excellent heat transfer efficiency, and superior mechanical strength [8,9]. It has been proved by Danaci et al. [10] that Ni/Al_2_O_3_ catalysts supported on 3D foam carriers significantly enhanced system heat transfer, achieving approximately 90% CO_2_ conversion at temperatures above 400 °C (only about 66% conversion for powder catalysts). The morphology support is proven to affect the catalytic performance of the structured catalyst. Watanabe et al. [11] compared integral structures loaded with Ni/CeO_2_ catalysts, such as helical, metal honeycomb, cordierite honeycomb, and traditional packed-bed structures. They found that the helical structure had a significantly higher CO_2_ conversion rate at 350 °C than the other structures (1.4–2 times) due to its promotional effect on the suppression of hot spots and the enhancement of mass transfer. Rauchenwald et al. [12] prepared SiOC-CH-Ni and SiOC-TBA-Ni catalysts with dendritic and prismatic pore structures respectively and found that the SiOC-CH-Ni facilitated the Ni dispersion and achieved a CO_2_ conversion rate of 69% at 400 °C, significantly outperforming the SiOC-TBA-Ni structure (39%). Kabakci et al. [13] used 3D printing to prepare Ni/Al_2_O_3_-EXT (extruded channels) and Ni/Al_2_O_3_-TW (twisted channels). Among them, the 0.5 mm Ni/Al_2_O_3_-TW enhanced mass transfer by virtue of the turbulent flow effect, achieving a CO_2_ conversion rate of 84% at 400 °C. It has also been verified that the chemical composition of the structural support affects the catalytic performance of structured catalysts. Italiano et al. [14] analyzed reactivity differences among Ni/CeO_2_-ZrO_2_/SiC and Ni/CeO_2_-ZrO_2_/Al_2_O_3_ open-cell foam structured catalysts and found that the SiC structure support can enhances temperature uniformity within the reaction system due to its high thermal conductivity. Consequently, the CO_2_ conversion of the SiC carbide foam ceramic catalyst was approximately 1.25 times that of the Al oxide foam ceramic catalyst under space velocity conditions of 47,027 h^−1^.

The strong metal–support interaction (SMSI) between Ni and the support exerts a significant influence on the performance of CO_2_ methanation. Studies have shown that a moderate SMSI can facilitate high dispersion of Ni active sites, suppress particle sintering, and optimize the formation and conversion pathways of key reaction intermediates (e.g., HCOO* and CO*), thereby enhancing both CO_2_ conversion and CH_4_ selectivity [15,16,17,18]. However, an excessively strong SMSI can induce the formation of inert metal–support compounds (e.g., NiAl_2_O_4_ spinel), diminish the exposure of active Ni sites, and overly constrain the electronic state of Ni particles along with their adsorption capacity for reactants, thereby ultimately suppressing the catalytic activity [19,20]. Most structural supports are inert, but they can also undergo solid-state reactions with the catalyst at high temperatures, which might induce the formation of strong interactions between the catalyst and structure support, subsequently affecting the catalytic activity of structured catalysts. In our recent work, we observed that the interfacial coupling linking the catalyst coating to the SiC substrate can regulate the binding strength of Ni and Al_2_O_3_, thus enhancing the initial catalytic activity of the catalyst. This interaction intensifies continuously during high-temperature reactions, which overly weakens the interaction between Ni and Al_2_O_3_ and ultimately results in a decline in the long-term stability of the catalyst [21]. Therefore, regulating the influence of structural supports on catalysts is crucial for achieving high-performance structured catalysts.

Relevant studies on Ni-based supported powder catalysts have demonstrated that the introduced third component can form SMSI with Ni species, which decrease the influence of supports on the catalyst [22,23,24,25,26]. Inspired by these findings, CeO_2_ was incorporated as a third component into the Ni/Al_2_O_3_/SiC structured catalyst to improve its catalytic performance. The catalytic performance test results show that CeO_2_ significantly boosts the CO_2_ methanation activity and stability of the Ni/Al_2_O_3_/SiC structured catalyst. To clarify the underlying mechanism, multiple characterization techniques including SEM, XRD, Raman, XPS, H_2_-TPR, H_2_-TPD, and CO_2_-TPD are employed. The aim of this work is to provide a viable strategy for suppressing the detrimental catalyst-support interactions in structured catalysts for CO_2_ methanation by leveraging CeO_2_-induced Ni-Ce interactions that simultaneously modulate Ni electronic states and reinforce the Al-Si interfacial bonding.

## 2. Experimental Section

### 2.1. Catalyst Synthesis and Preparation

#### 2.1.1. Pretreatment of Porous Sic Ceramic Supports

Porous SiC ceramics (abbreviated as SiC hereafter) were fabricated via the particle stacking sintering approach, with the full synthesis protocol detailed in our previously reported work [27]. To eliminate loosely attached SiC particles from the substrate surface, the as-synthesized porous SiC ceramics were subjected to ultrasonic cleaning prior to catalyst loading.

#### 2.1.2. Fabrication of Ni-xCeO_2_/Al_2_O_3_/SiC Structured Catalysts

All Ni-xCeO_2_/Al_2_O_3_/SiC structured catalysts were synthesized via a two-step vacuum impregnation route. In the first step, the pre-treated porous SiC supports were immersed in aluminum precursor solution, followed by drying at 120 °C for 7 h and calcination at 600 °C for 2 h to form a stable Al_2_O_3_ coating on the SiC surface. In the subsequent step, the Al_2_O_3_-modified SiC supports were soaked in a mixed precursor solution containing nickel and cerium species for 6 h, then dried at 120 °C for 7 h and calcined at 400 °C for 3 h to obtain the final catalysts. The as-prepared samples were designated as Ni-xCeO_2_/Al_2_O_3_/SiC, where x represents the mass fraction of CeO_2_ in the active layer (x = 10, 14, 16, 18). For control experiments, the Ni/Al_2_O_3_/SiC structured catalyst was synthesized via an identical procedure without the addition of a cerium precursor. The detailed actual component loadings of all catalysts, including Ni, CeO_2_, Al_2_O_3_ and total active layer loadings, are provided in Table S1. Ni loading was strictly controlled at 13.0 wt% for all samples.

### 2.2. Material Physicochemical Characterization

Field emission scanning electron microscopy (SEM, Hitachi S-4800) coupled with energy dispersive X-ray spectroscopy (EDS) was employed to characterize the surface morphology and elemental distribution of all as-prepared samples. Crystal phase analysis was performed via X-ray diffraction (XRD) on a Rigaku MiniFlex 600X diffractometer with Cu Kα radiation, with a 2θ scanning range of 10° to 80° and a scanning speed of 15° min^−1^. Raman spectra were collected on a LabRAM HR800 spectrometer with a 532 nm excitation laser, covering a wavenumber range of 100–700 cm^−1^. Fourier transform infrared (FT-IR) spectra were recorded on a Thermo Nicolet 8700 spectrometer using the KBr pellet method, with a testing range of 400–4000 cm^−1^. X-ray photoelectron spectroscopy (XPS, Thermo Scientific (Waltham, MA, USA) K-Alpha, Al Kα radiation at 1486.6 eV) was conducted to analyze the chemical states of surface elements on the catalyst surface.

The reduction behavior and adsorption properties of the catalysts toward H_2_ and CO_2_ were investigated using temperature-programmed techniques. In a typical H_2_-TPR experiment, 0.05 g of the sample was first pretreated at 150 °C for 1 h under a flow of 10 vol% He/Ar to remove any adsorbed impurities. After cooling to 55 °C, the temperature was raised to 800 °C at a ramp of 10 °C·min^−1^ under a 10 vol% H_2_/Ar atmosphere, and the hydrogen consumption was continuously recorded by a thermal conductivity detector (TCD). For the H_2_-TPD and CO_2_-TPD measurements, 0.1 g of the catalyst was reduced in situ at 300 °C for 1 h in a 10 vol% H_2_/Ar stream. Subsequently, the sample was cooled to 55 °C and exposed to pure H_2_ or pure CO_2_ for 1 h to achieve saturation adsorption. After purging with high-purity He or Ar to eliminate physisorbed species, the desorbed amounts of H_2_ and CO_2_ were monitored by TCD while heating to 800 °C at 10 °C·min^−1^.

### 2.3. Catalytic Performance Evaluation

Catalytic performance tests were carried out in a fixed-bed reactor system, which consists of a tubular furnace fitted with a precision temperature controller. To assess the catalytic performance of Ni-xCeO_2_/Al_2_O_3_/SiC structured catalysts with different CeO_2_ loading amounts, the catalysts were crushed and loaded into the isothermal zone of the reactor tube. A K-type thermocouple was inserted directly into the catalyst bed to monitor, regulate and maintain a stable reaction temperature throughout the test.

All catalytic tests were performed under atmospheric pressure (0.1 MPa), with a total feed gas flow rate of 60 mL·min^−1^ (volume ratio of H_2_/N_2_/CO_2_ = 60/25/15). A constant catalyst mass of 0.1 g was used for all activity tests to ensure consistent comparison. The reaction temperature was set from 200 °C to 400 °C with an interval of 50 °C for each test point. Before initiating the formal catalytic testing, all as-prepared catalysts underwent in situ reduction treatment under a 10 vol% H_2_/Ar gas stream (flow rate: 60 mL·min^−1^), which was maintained at 400 °C for a duration of 2 h. A cold trap was installed at the reactor outlet to remove gaseous water generated from the methanation reaction, and the dried outlet gas was introduced into an online gas chromatograph (GC 9860, NetChrom VER.01.2022.05.20) for quantitative component analysis. All experiments were repeated three times, and the relative standard deviation of the results was less than 5%, indicating good reproducibility. The CO_2_ conversion ($X_{CO_{2}}$), CH_4_ selectivity (*S_CH_*_4_) and CH_4_ specific reaction rate (*R_CH_*_4_) were respectively quantified using the Formulas (1)–(3) presented below:

(1) $$X_{CO_{2}}\;=\;\frac{V_{{\mathrm{CO}}_{2},\mathrm{in}}\;-{\;V}_{{\mathrm{CO}}_{2},\mathrm{out}}}{V_{CO_{2},\mathrm{in}}}\;\times \;100\%$$

(2) $$S_{CH_{4}}=\frac{V_{CH_{4}}}{V_{{\mathrm{CO}}_{2},\mathrm{in}}-V_{{\mathrm{CO}}_{2},\mathrm{out}}}\;\times \;100\%$$

(3) $$R_{CH_{4}}=\frac{X_{CO_{2}}\cdot V_{CO_{2},\mathrm{in}}\cdot S_{CH_{4}}}{m_{\mathrm{cat}}\cdot V_{m}\cdot 60000}$$

To further evaluate the intrinsic catalytic activity of Ni active sites and eliminate the interference of catalyst composition differences caused by CeO_2_ addition, the CH_4_ specific reaction rate normalized by Ni mass ($R_{{\mathrm{CH}}_{4},\mathrm{Ni}}$) was additionally calculated using Formula (4):

(4) $$R_{{\mathrm{CH}}_{4},\mathrm{Ni}}\;=\;\frac{R_{{\mathrm{CH}}_{4},\mathrm{total}}}{\omega_{\mathrm{Ni}}}$$

In the above equations, *V_in_* and *V_out_* refer to the volumetric flow velocities of the feed and effluent gases (unit: mL·min^−1^), *m_cat_* is the mass of the tested catalyst (unit: g), and *V_m_* is the molar volume of ideal gas under standard conditions (22.4 L·mol^−1^), and ($\omega_{\mathrm{Ni}}$) is the constant mass fraction of Ni in the total catalyst, which was strictly controlled at 13.0 wt% for all prepared samples to ensure a consistent baseline for activity comparison.

## 3. Results and Discussion

### 3.1. Structure and Morphology Analysis

**Figure 1:**
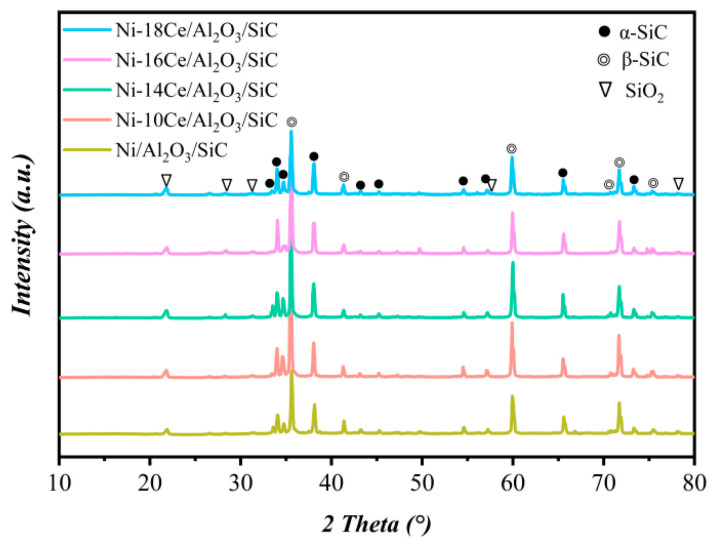
XRD patterns of Ni/Al_2_O_3_/SiC, Ni-10CeO_2_/Al_2_O_3_/SiC, Ni-14CeO_2_/Al_2_O_3_/SiC, Ni-16CeO_2_/Al_2_O_3_/SiC, and Ni-18CeO_2_/Al_2_O_3_/SiC catalysts.

The phase composition of the Ni/Al_2_O_3_/SiC and Ce-modified Ni/Al_2_O_3_/SiC samples was first analyzed via XRD analysis. As shown in Figure 1, all prepared samples exhibited highly crystalline characteristic peaks corresponding respectively to the *α*-SiC (PDF#49-1428), *β*-SiC (PDF#29-1129), and SiO_2_ (PDF#39-1425) phases [28,29]. Owing to the low loading content of the active catalyst layer and the obscuring effect on diffraction signals caused by the highly crystalline SiC support, the characteristic diffraction peaks corresponding to NiO, *γ*-Al_2_O_3_, and CeO_2_ were not clearly observed in the XRD patterns.

**Figure 2:**
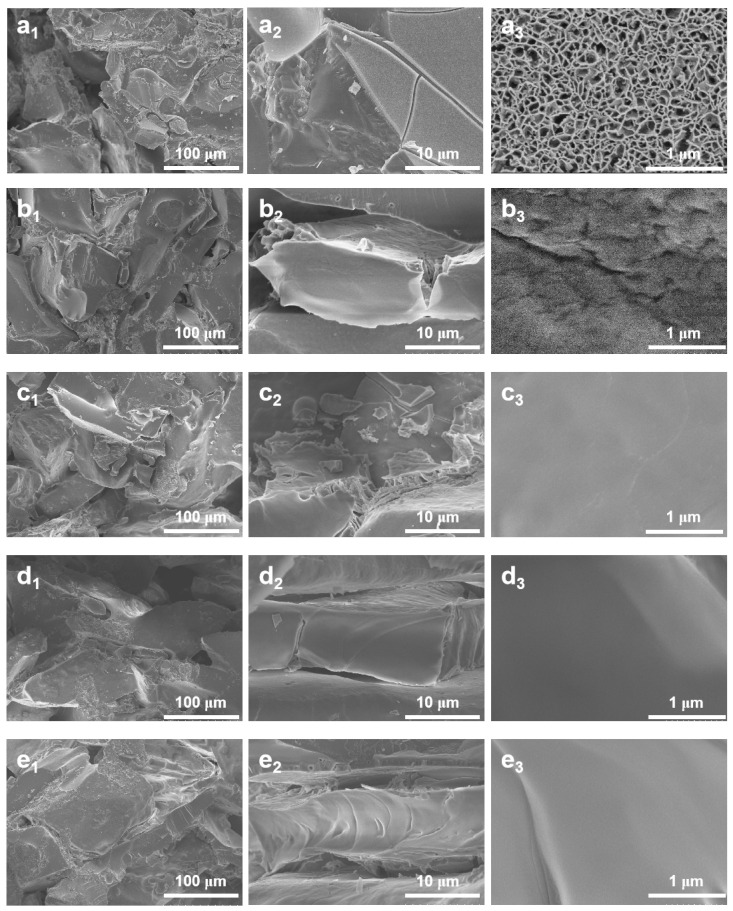
SEM images of Ni/Al_2_O_3_/SiC (**a_1_**–**a_3_**), Ni-10CeO_2_/Al_2_O_3_/SiC (**b_1_**–**b_3_**), Ni-14CeO_2_/Al_2_O_3_/SiC (**c_1_**–**c_3_**), Ni-16CeO_2_/Al_2_O_3_/SiC (**d_1_**–**d_3_**), and Ni-18CeO_2_/Al_2_O_3_/SiC (**e_1_**–**e_3_**) catalysts.

SEM observations (Figure 2) reveal that the unmodified Ni/Al_2_O_3_/SiC catalyst (Figure 2(a_1_–a_3_)) features a block-like catalyst layer loaded on SiC channels, with obvious cracks arising from a thermal expansion coefficient mismatch between the Ni-Al catalyst layer and the SiC support [30]. In contrast, the Ce-modified Ni-xCeO_2_/Al_2_O_3_/SiC catalysts (Figure 2(b_1_–e_3_)) retain the block-like loading morphology while exhibiting a significantly smoother and crack-free surface. These results confirmed that Ce modification modulates the interfacial bonding state between the catalyst layer and SiC support. EDS analysis (Figure S1) clearly shows the elemental distribution. A distinct boundary between the SiC support and the catalyst layer is marked by the separate regions of Si and Al. The intensity of the Ce signal varies systematically with its intended modification amount, confirming the controlled incorporation of CeO_2_. Moreover, both Ni and Ce are uniformly dispersed across the catalyst surface. This uniform distribution creates favorable structural conditions for interfacial interaction. Based on these observations, we hypothesize that the formation of Ni-Ce interaction structures may alleviate thermal stress, effectively reducing structural defects and enhancing interfacial adhesion. Further microscopic evidence is needed to confirm this [31,32].

### 3.2. CO_2_ Methanation Activity and Stability

**Figure 3:**
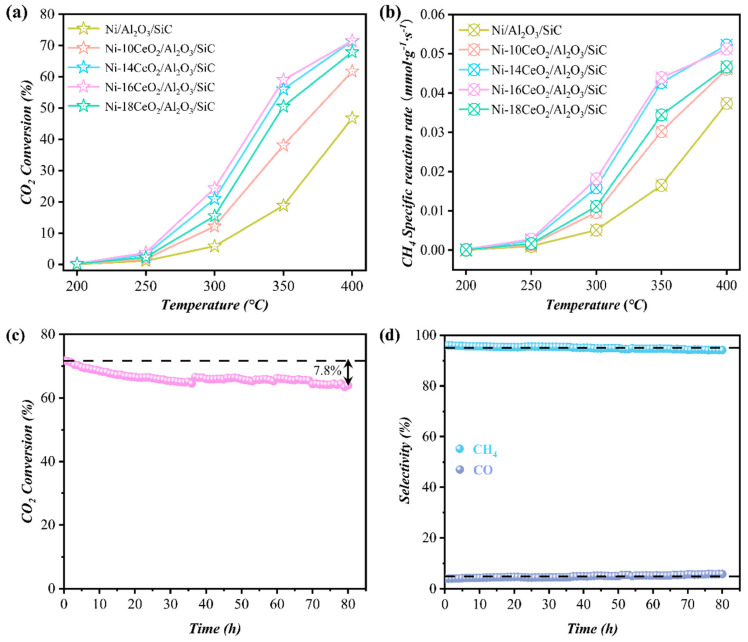
CO_2_ conversion (**a**) and CH_4_ specific reaction rate (**b**) of Ni-xCeO_2_/Al_2_O_3_/SiC catalysts at different temperatures: 1440 h^−1^, CO_2_ conversion (**c**), and selectivity (**d**) of Ni-16CeO_2_/Al_2_O_3_/SiC catalyst at 400 °C.

Figure 3a,b presents the CO_2_ conversion rate and CH_4_ specific reaction rate of the structured Ni/Al_2_O_3_/SiC and Ni-xCeO_2_/Al_2_O_3_/SiC catalysts. Ni/Al_2_O_3_/SiC shows poor catalytic function in the methanation of CO_2_, with the highest CO_2_ conversion of 46.8% and a specific CH_4_ production rate of 0.037 mmol·g^−1^·s^−1^ at 400 °C. All Ni-xCeO_2_/Al_2_O_3_/SiC catalysts presented a higher rate than that of the Ni/Al_2_O_3_/SiC catalyst, which indicates that CeO_2_ modification can effectively enhance the catalyst activity. Among these Ni-xCeO_2_/Al_2_O_3_/SiC catalysts, the optimal Ni-16CeO_2_/Al_2_O_3_/SiC catalyst displayed a CO_2_ conversion of 71.6% at 400 °C, which is approximately 1.5 times that of the Ni/Al_2_O_3_/SiC catalyst. Overall, Ce modification can significantly enhance the activity of the Ni/Al_2_O_3_/SiC structured catalyst, corresponding to a total CH_4_ specific reaction rate of 0.056 mmol·g^−1^·s^−1^. After normalization by the constant Ni mass, its ($R_{{\mathrm{CH}}_{4},\mathrm{Ni}}$) reaches 0.431 mmol·$g_{\mathrm{Ni}}^{-1}$·s^−1^, 1.51 times higher than that of Ni/Al_2_O_3_/SiC (0.285 mmol·$g_{\mathrm{Ni}}^{-1}$·s^−1^).

The durability during prolonged operation of the Ni-16CeO_2_/Al_2_O_3_/SiC catalyst, a core prerequisite for industrial application, was evaluated via an 80 h test. The catalyst demonstrated outstanding stability as its CO_2_ conversion decreased only slightly from 71.6% to 63.8%, representing a mere 7.8% loss. Meanwhile, the CH_4_ selectivity remained highly stable at approximately 95% without significant fluctuation. This performance is superior to that of the Ni/Al_2_O_3_/SiC catalyst, which exhibited a larger decrease in CO_2_ conversion (~15%) under similar conditions [21]. These results indicate that CeO_2_ modification effectively enhances long-term operational stability. As summarized in Table S3, our catalyst surpasses powder catalysts in durability and matches advanced SiC-structured counterparts. Such favorable stability originates from CeO_2_-induced structural stabilization that strengthens interfacial adhesion and suppresses unfavorable interfacial evolution during long-term reaction. Post-reaction characterization of the spent Ni-16CeO_2_/Al_2_O_3_/SiC catalyst further corroborates this conclusion: XRD analysis detected no peak shifts or new crystalline phases after the 80 h test (Figure S2), while SEM observation confirmed that the coating morphology remained intact without noticeable crack propagation (Figure S3).

Previous studies have confirmed that during the prolonged operation of the Ni/Al_2_O_3_/SiC catalyst, the Al-Si interaction between the support and the catalyst layer is progressively enhanced. This strengthening attenuates the Ni-Al interaction, ultimately leading to catalyst deactivation [21]. The SEM results in this study demonstrate that CeO_2_ modification may have promoted the interfacial adhesion between the catalyst layer and the SiC support while concurrently intensifying the Al-Si interaction. Nevertheless, the Ni-16CeO_2_/Al_2_O_3_/SiC catalyst exhibits superior catalytic stability during an 80 h longevity test. This finding indicates that the influence of variations in the Al-Si interaction on the performance of the modified catalyst has been substantially mitigated. Consequently, CeO_2_ modification effectively suppresses the detrimental impact of evolving Al-Si interactions on the CO_2_ methanation performance of Ni-based catalysts. In the following text, the characterizations of Ni/Al_2_O_3_/SiC and Ni-16CeO_2_/Al_2_O_3_/SiC were performed to analyze the influence of Ce modification.

### 3.3. Surface Composition and Physicochemical Properties

**Figure 4:**
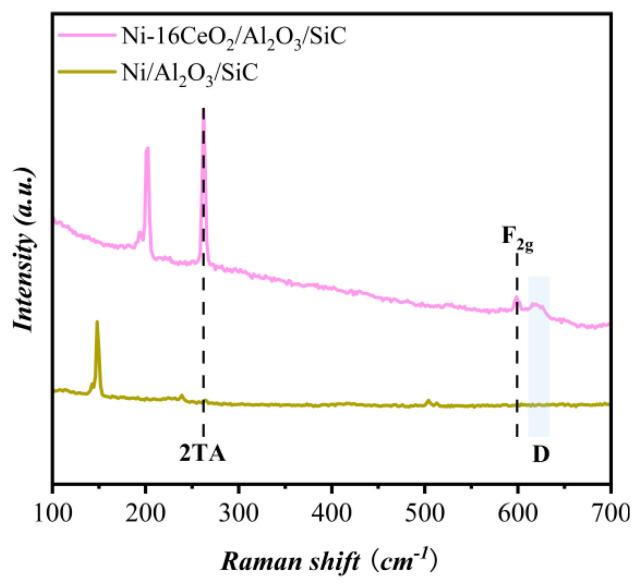
Raman spectra of Ni/Al_2_O_3_/SiC, and Ni-16CeO_2_/Al_2_O_3_/SiC catalysts.

Raman spectroscopy was conducted for an in-depth investigation into the changes in chemical bonds on the catalyst surface. As shown in Figure 4, distinct characteristic peaks of the CeO_2_ phase appeared on the catalyst surface of Ni/Al_2_O_3_/SiC catalyst after CeO_2_ modification. These characteristic peaks respectively correspond to the lattice vibrations of the face-centered cubic structure (F_2_g), vibrations induced by oxygen vacancies (D), and second-order transverse acoustic vibrations (2TA) [33]. Compared with the characteristic peaks of CeO_2_ in the literature, the peaks of this catalyst showed a significant blue shift, which indicates an increase in the disorder of the crystal structure and an increase in the number of defect sites in the catalyst [34]. The lattice distortion and increase in defects revealed by the blue shift were further confirmed in the detailed analysis of the oxygen-vacancy-characteristic D peak. Jiang et al. [35] conducted a detailed classification of the D peak, dividing it into three peaks of D_1_ peak (580~620 cm^−1^), D_2_ (620~640 cm^−1^), and D_3_ (540~570 cm^−1^). The D_1_ peak mainly results from the vibration when oxygen atoms migrate to the octahedral interstitial positions of CeO_2_. The D_2_ peak is closely related to extrinsic defects caused by foreign-element doping, while the D_3_ peak contains both types of the above-mentioned defects. For the Ni-16CeO_2_/Al_2_O_3_/SiC structured catalyst, its D peak is in the range of 611-634 cm^−1^, indicating that the incorporation of foreign elements into the CeO_2_ lattice is the main cause of the formation of oxygen vacancies.

**Figure 5:**
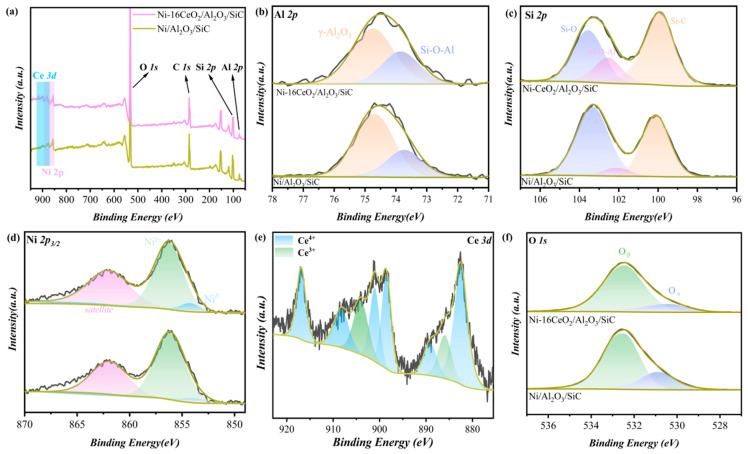
XPS spectra of Ni/Al_2_O_3_/SiC and Ni-16CeO_2_/Al_2_O_3_/SiC catalysts: full spectrum (**a**), Al *2p* (**b**), Si *2p* (**c**), Ni *2p* (**d**), Ce *3d* (**e**), and O *1s* (**f**).

XPS analysis was performed to study the effect of CeO_2_ modification on the surface component of the catalyst. Figure 5a shows that Ce has been successfully introduced into the catalyst. In the Al *2p* spectrum (Figure 5b), peaks at around 74.70 eV and 73.72 eV are assigned to *γ*-Al_2_O_3_ and the interfacial Si-O-Al bond respectively [36,37,38]. After CeO_2_ modification, the relative peak-area ratio of the Si-O-Al bond increased significantly from 0.38 to 0.52 (Table S1). This shows that the introduction of CeO_2_ promotes and stabilizes the chemical bonding between the Al_2_O_3_ coating and the SiC support, thereby strengthening the Al-Si interaction. The Si *2p* spectra (Figure 5c) further validate these interfacial changes (the relative area ratio of the peak (about 102.10 eV) corresponding to the interfacial Si-O-Al bond increased from 0.11 to 0.31 (Table S1)) [39]. After CeO_2_ modification, this ratio rose substantially (Table S1). This result clearly indicates that CeO_2_ effectively enhances the interfacial coupling between Al_2_O_3_ and the SiC support.

From Figure 5d, the Ni *2p* spectrum exhibits distinct peaks at 861.94 eV, 856.17 eV, and 853.84 eV, corresponding to the satellite peak, Ni^2+^ state, and Ni^δ+^ state respectively [40,41]. After CeO_2_ modification, the binding energy of the main Ni^2+^ peak shifts negatively to 856.13 eV. Also, the signal intensity of Ni^δ+^ gradually increases. This indicates that the electron density of Ni species has increased due to the newly formed Ni-Ce interaction. This interaction effectively weakens the originally excessive interaction between Ni and Al_2_O_3_. Meanwhile, the presence of Ce^3+^ in the Ce *3d* spectrum confirms the generation of oxygen vacancies. These vacancies act as electron donors, further optimizing the electronic state of Ni sites [35]. The change in the O *1s* spectrum (Figure 5f) provides information about surface oxygen species. The O_β_ peak at around 531.3 eV can be attributed to surface-adsorbed oxygen species [42,43]. After CeO_2_ modification, the relative proportion of O_β_ species increased from 76% to 82%. Notably, oxygen vacancies in oxides have three charge states: positively charged F^2+^, single-electron F^+^, and neutral F^0^ [44]. In CeO_2_, the Ce^4+^/Ce^3+^ redox couple traps electrons from oxygen vacancy formation, so F^2+^ vacancies dominate in our catalysts, consistent with XPS results. These F^2+^ vacancies enhance CO_2_ adsorption and, together with Ce^3+^, promote C=O bond cleavage. In summary, XPS reveals that CeO_2_ modification stabilizes the support-coating architecture by enhancing Al-Si interfacial bonding while simultaneously tuning the electronic density of Ni through Ni-Ce interaction. Consistent with the XPS finding of enhanced Ni electronic density, CO pulse chemisorption measurements further showed that CeO_2_ modification increased the Ni dispersion from 0.74% to 0.85% (Figure S4 and Table S4), suggesting that the modulated Ni-Ce interaction helps improve the accessibility of Ni active sites.

**Figure 6:**
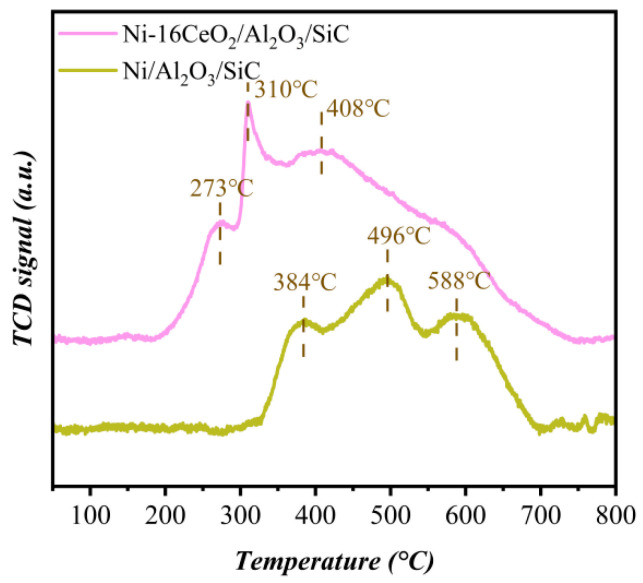
H_2_-TPR profiles of Ni/Al_2_O_3_/SiC, and Ni-16CeO_2_/Al_2_O_3_/SiC catalysts.

H_2_-TPR was conducted to examine the metal–support interaction and reducibility of the catalysts. As shown in Figure 6, broad reduction peaks can be found at 200–700 °C in the H_2_-TPR profiles. For the Ni/Al_2_O_3_/SiC catalyst, three reduction peaks of highly reducible NiO species, NiO with moderate metal–support interaction, and Ni^2+^ species strongly interacting with the support are observed at 384 °C, 496 °C, and 588 °C, respectively [42,45,46]. Upon modification with CeO_2_, all reduction peaks for the Ni-16CeO_2_/Al_2_O_3_/SiC catalyst shift to significantly lower temperatures (273 °C, 310 °C, and 408 °C, respectively). The pronounced shift in reduction peaks originates from the altered metal–support interactions induced by CeO_2_. Specifically, the introduction of CeO_2_ promotes the Ce^4+^/Ce^3+^ redox cycles, which facilitate the formation of oxygen vacancies. These vacancies act as electron-transfer mediators, increasing the electron density of Ni species and thereby weakening the Ni-O bonds. This electronic effect attenuates the originally strong Ni-Al interaction while concurrently enhancing the Ni-Ce interaction, which collectively lowers the activation energy for reduction and significantly improves the reducibility and low-temperature activity of Ni species [47,48].

**Figure 7:**
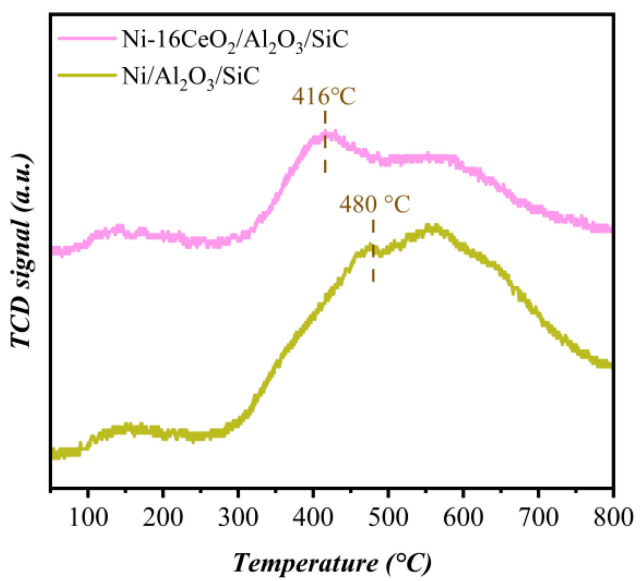
H_2_-TPD profiles of Ni/Al_2_O_3_/SiC, and Ni-16CeO_2_/Al_2_O_3_/SiC catalysts.

H_2_-TPD reveals the adsorption strength and site types of H_2_ on the catalyst. Both catalysts exhibited desorption peaks in the temperature range of 250–800 °C (Figure 7). For Ni/Al_2_O_3_/SiC, the moderately strong desorption peak at 480 °C corresponds to the active H species adsorbed on the Ni surface, and these H_2_ are key active intermediates in the CO_2_ methanation reaction. The strong desorption peak near 540 °C is attributed to the desorption process of strongly chemisorbed hydrogen [35,46]. Upon CeO_2_ modification, the moderately strong adsorption peak for the Ni-16CeO_2_/Al_2_O_3_/SiC catalyst shifts markedly to a lower temperature of 416 °C, accompanied by a substantial increase in peak area. In contrast, the position of the strong chemisorption peak remains largely unchanged. The pronounced shift and enhanced area of the moderate-strength peak indicate that the hydrogen adsorption/desorption behavior on Ni sites has been remarkably enhanced. The strong Ni-Ce interaction increases the electron density of Ni species and promotes the formation of surface oxygen vacancies, as shown by XPS and Raman spectroscopy. Both effects collectively weaken the Ni-H bonding strength and increase the number of available adsorption sites for activated hydrogen. Meanwhile, the invariance of the high-temperature peak indicates that the intrinsic adsorption characteristics of the Al_2_O_3_/SiC support remain largely unaltered, confirming that CeO_2_ modification scarcely changes the lattice features of the support itself [47,48]. This further underscores that the adsorption modulation occurs predominantly at the Ni active sites influenced by CeO_2_.

**Figure 8:**
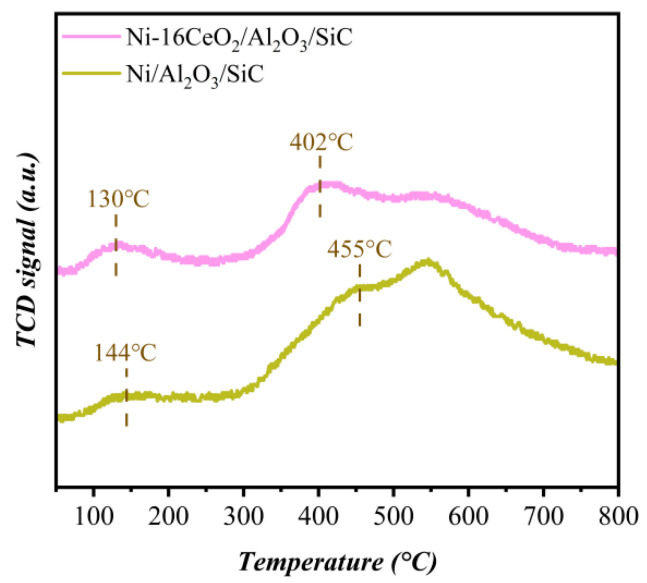
CO_2_-TPD profiles of Ni/Al_2_O_3_/SiC, and Ni-16CeO_2_/Al_2_O_3_/SiC catalysts.

CO_2_-TPD was performed to evaluate the surface basicity and CO_2_ adsorption/activation properties (Figure 8). The peak at 144 °C is related to the weak basic sites of the catalyst, which may result from the physical adsorption of CO_2_ as well as the adsorption on surface hydroxyl groups and oxygen vacancies. The peak at ~455 °C corresponds to CO_2_ desorbed from moderately strong basic sites, typically associated with bidentate carbonate (B-CO_3_*) species formed on metal-oxygen pairs [46,49]. After CeO_2_ modification, the Ni-16CeO_2_/Al_2_O_3_/SiC catalyst shows a notable increase in the peak area at 130 °C and the peak for moderately strong basic sites shifts to a significantly lower temperature (402 °C) with a substantially enlarged area. These changes are attributed to the strong Ni-Ce interaction and the accompanying increase in oxygen-vacancy concentration (as revealed by XPS and Raman spectroscopy), which offer additional weak basic sites for CO_2_ adsorption [33]. Concurrently, the attenuation of the Ni-Al interaction optimizes the electronic density of surface Ni species, which facilitates the formation and stabilization of bidentate carbonate intermediates, thereby strengthening the moderate basicity and lowering its desorption energy.

### 3.4. The Proposed Promotional Mechanism Analysis

**Figure 9:**
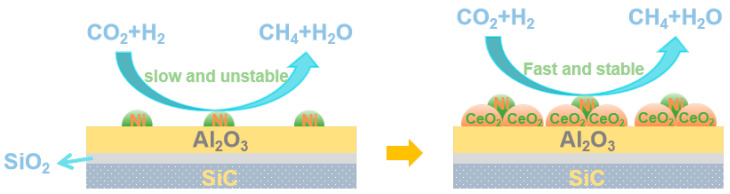
Schematic Diagram of the Promotion Mechanism of CeO_2_ Modification on CO_2_ Methanation over Ni/Al_2_O_3_/SiC Structured Catalysts.

Based on the above-mentioned characterization results, CeO_2_ modification significantly enhances the CO_2_ methanation performance of Ni/Al_2_O_3_/SiC catalysts by synergistically optimizing their multi-level properties. From a macroscopic perspective, the SEM results show that CeO_2_ modification effectively reduces the cracks in the catalyst layer, enhancing the structural integrity of the coating. This structural optimization corresponds to significant changes in the bulk reduction properties and surface chemical states of the catalyst. The H_2_-TPR reduction test confirms a substantial decrease in the reduction temperature of Ni species, and the XPS and Raman analyses further reveal the generation of a large number of oxygen vacancies on the catalyst surface and an increase in the electron density of Ni species. Subsequently, the optimized surface properties directly affect the adsorption behavior of reactants. The H_2_-TPD and CO_2_-TPD results indicate a significant increase in the adsorption sites of active hydrogen and moderately strong CO_2_ adsorption sites on the catalyst surface. Ultimately, the comprehensive improvements in the structural stability of the catalyst, reducibility of active sites, and reactant adsorption and activation capabilities jointly promote the simultaneous enhancement of its low-temperature activity and long-term stability. The catalytic mechanism schematic diagram in Figure 9 visually compares the reaction differences before and after CeO_2_ modification. In the unmodified catalyst, the enhanced interaction between Ni and Al_2_O_3_ restricts the reaction process, manifested as a slow CO_2_ methanation reaction and poor stability. However, CeO_2_ modification significantly strengthens the Al-Si interaction, weakens the interaction between Ni and Al_2_O_3_, and the enhanced interaction between the formed Ni and Ce species and the abundant oxygen vacancies establishes an efficient reaction pathway, enabling the rapid conversion of CO_2_ and H_2_ and the stable progress of the reaction.

## 4. Conclusions

In summary, this study has significantly enhanced the CO_2_ methanation performance of Ni/Al_2_O_3_/SiC structured catalysts by regulating their interfacial interactions and surface physicochemical properties through CeO_2_ modification. The Ni-16CeO_2_/Al_2_O_3_/SiC catalyst exhibits the optimal catalytic performance. At 400 °C, the CO_2_ conversion rate can reach 71.6%, approximately 1.5 times that of the unmodified Ni/Al_2_O_3_/SiC catalyst. This catalyst also possesses excellent long-term stability. During the 80 h continuous test, the CO_2_ conversion rate loss is only 7.8%, and the CH_4_ selectivity remains above 95%. The improvement in catalyst performance stems from multiple synergistic effects brought about by CeO_2_ modification. Specifically, the strong interaction formed between CeO_2_ and Ni species effectively weakens the overly strong metal–support interaction between Ni and Al_2_O_3_, promoting the reduction in Ni species. Meanwhile, the oxygen storage-release capacity of CeO_2_ induces the generation of a large number of oxygen vacancies on the catalyst surface, optimizes the distribution of moderately basic sites on the catalyst surface, and significantly enhances the adsorption and activation efficiency of H_2_ and CO_2_. This study has clarified the regulatory mechanism of CeO_2_ modification on the CO_2_ methanation performance of Ni-based structured catalysts, providing a theoretical basis and feasible strategy for the design and development of highly efficient structured catalysts.

## Data Availability

The original contributions presented in this study are included in the article/Supplementary Material. Further inquiries can be directed to the corresponding author.

## Supplementary Materials

The following supporting information can be downloaded at: https://www.mdpi.com/article/10.3390/ma19122612/s1, Figure S1. EDS-mapping images of Ni/Al_2_O_3_/SiC (a1–a6), Ni-10CeO_2_/Al_2_O_3_/SiC (b1–b6), Ni-14CeO_2_/Al_2_O_3_/SiC (c1–c6), Ni-16CeO_2_/Al_2_O_3_/SiC (d1–d6), and Ni-18CeO_2_/Al_2_O_3_/SiC (e1–e6) catalyst. Figure S2. XRD images of the Ni-16CeO_2_/Al_2_O_3_/SiC catalyst after the 80 h continuous test and the fresh catalyst. Figure S3. SEM images of Ni/Al_2_O_3_/SiC (a) and Ni-16CeO_2_/Al_2_O_3_/SiC (b) catalyst after the 80 h continuous. Figure S4. CO pulse chemisorption profiles of Ni/Al_2_O_3_/SiC and Ni-16CeO_2_/Al_2_O_3_/SiC catalysts. Table S1. Specific catalyst loadings of Ni/Al_2_O_3_/SiC and Ni-xCeO_2_/Al_2_O_3_/SiC catalysts. Table S2. Changes in the relative area ratio of the Si-O-Al bond in the Al 2p and Si 2p XPS spectra of the Ni/Al_2_O_3_/SiC and Ni-16CeO_2_/Al_2_O_3_/SiC catalysts. Table S3. Performance comparison of representative Ni-based CO_2_ methanation catalysts. Table S4. CO pulse chemisorption results, metal dispersion, and crystallite size of Ni-16CeO_2_/Al_2_O_3_/SiC and Ni/Al_2_O_3_/SiC catalysts.
