# Supplementary material for: CeO2 Induced Ni-Ce Interaction Enables Efficient CO2 Methanation on Ni/Al2O3/SiC Structured Catalyst
Source: Materials (Basel). 2026 Jun 17;19(12):2612. doi: 10.3390/ma19122612 (PMC13304082; doi:10.3390/ma19122612)
Supplement: Supplementary file 1 [file materials-19-02612-s001.zip › materials-4331140-supplementary.pdf]

Supplementary Materials

CeO_2 induced Ni-Ce interaction enables efficient CO_2 methanation on Ni/Al_2O_3/SiC structured catalyst

Jiyue Xu[a], Jiaxin Qian[a], Xiangli Liu[a], Fei Gao[a, b], Yiqing Zeng[a, b, *], Shule Zhang[c], Zhaoxiang Zhong[a, b, d]

[a] *School of Environmental Science and Engineering, Nanjing Tech University, Nanjing 211816, PR China*

[b] *State Key Laboratory of Materials-Oriented Chemical Engineering, National Engineering Research Center for Special Separation Membrane, Nanjing Tech University, Nanjing 211816, PR China*

[c] *School of Chemistry and Chemical Engineering, Nanjing University of Science and Technology, Nanjing 210094, PR China*

[d] *NJTECH University Suzhou Future Membrane Technology Innovation Center, Suzhou, 215300, PR China*

** Corresponding authors' E-mail: yiqingzeng@163.com (Yiqing Zeng)*

Figures

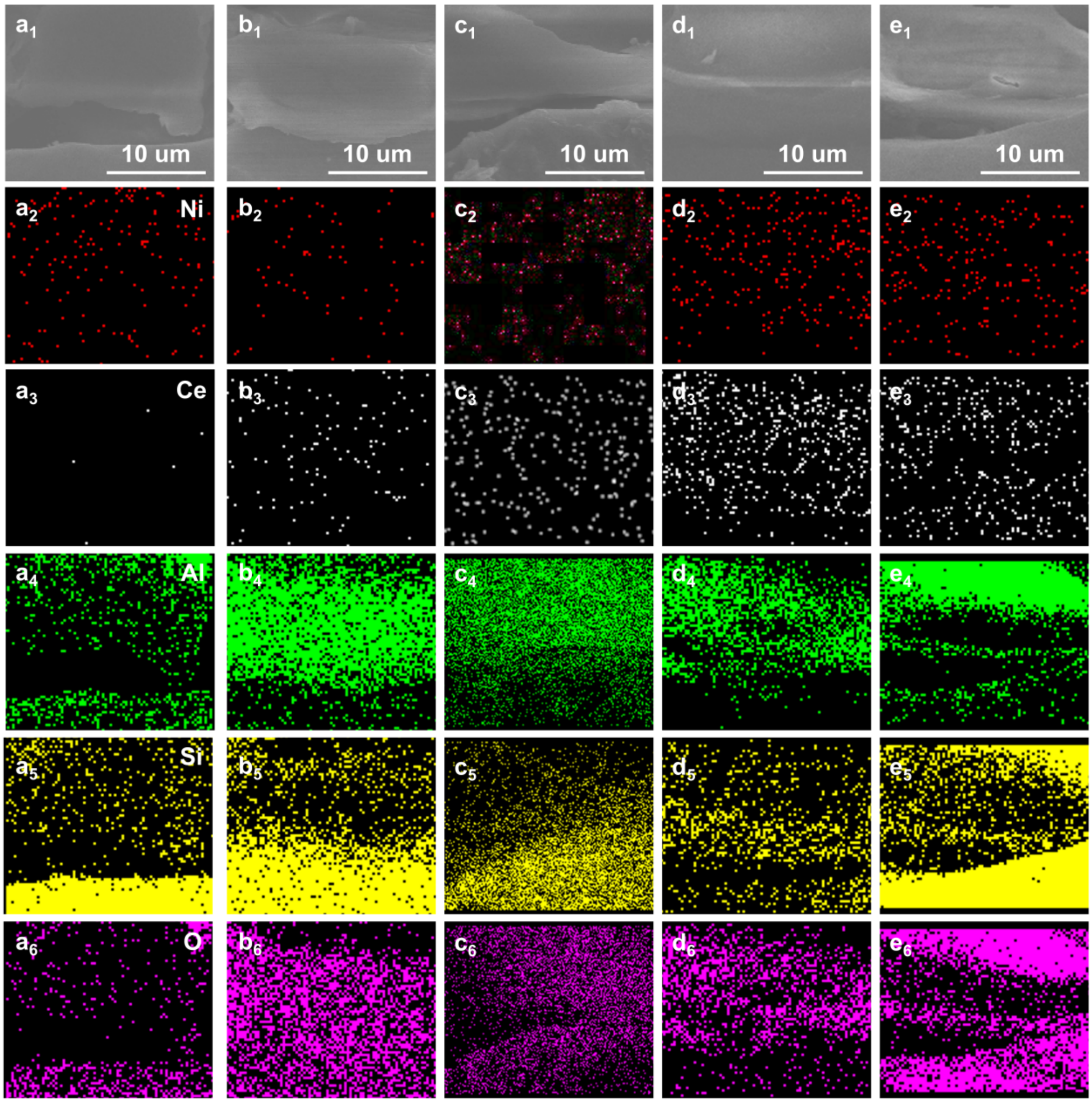

Figure S1 EDS-mapping images of Ni/Al_2O_3/SiC (a1-a6), Ni-10CeO_2/Al_2O_3/SiC (b1-b6), Ni-14CeO_2/Al_2O_3/SiC (c1-c6), Ni-16CeO_2/Al_2O_3/SiC (d1-d6), and Ni-18CeO_2/Al_2O_3/SiC (e1-e6) catalyst.

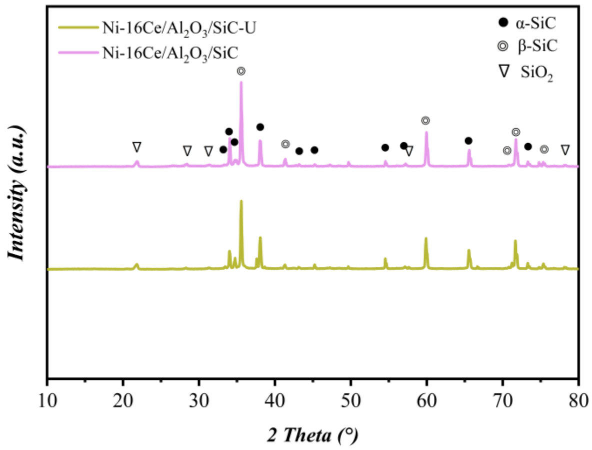

Figure S2 XRD images of the Ni-16CeO$_2$/Al$_2$O$_3$/SiC catalyst after the 80 h continuous test and the fresh catalyst

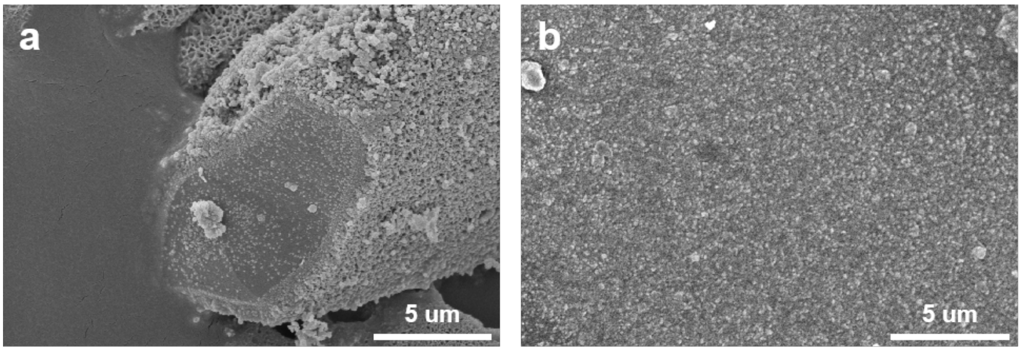

Figure S3 SEM images of Ni/Al$_2$O$_3$/SiC (a) and Ni-16CeO$_2$/Al$_2$O$_3$/SiC (b) catalyst after the 80 h continuous

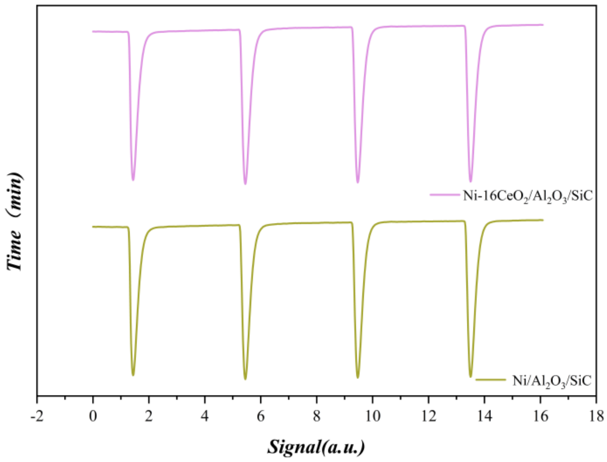

Figure S4 CO pulse chemisorption profiles of Ni/Al$_2$O$_3$/SiC and Ni-16CeO$_2$/Al$_2$O$_3$/SiC catalysts.

Table

Table S1 Specific catalyst loadings of $Ni/Al_2O_3/SiC$ and $Ni-xCeO_2/Al_2O_3/SiC$ catalysts

**Catalyst**	**Ni loading /g**	**CeO_2 loading /g**	**Al_2O_3 loading /g**	**Total active layer loading / g**	**Ni mass fraction (total catalyst) / wt%**	**CeO_2 mass fraction (active layer) / wt%**
$Ni/Al_2O_3/SiC$	0.013	0	0.0634	0.0764	13.0	0
$Ni-10CeO_2/Al_2O_3/SiC$	0.013	0.0087	0.0629	0.0846	13.0	10.3
$Ni-14CeO_2/Al_2O_3/SiC$	0.013	0.0127	0.0624	0.0881	13.0	14.4
$Ni-16CeO_2/Al_2O_3/SiC$	0.013	0.0147	0.0624	0.0901	13.0	16.3
$Ni-18CeO_2/Al_2O_3/SiC$	0.013	0.0166	0.0626	0.0922	13.0	18.0

Table S2 Changes in the relative area ratio of the Si-O-Al bond in the Al 2p and Si 2p XPS spectra of the $Ni/Al_2O_3/SiC$ and $Ni-16CeO_2/ Al_2O_3/SiC$ catalysts

**Sample**	**Proportion of Si-O-Al Peak Area in Al 2p Spectrum**	**Proportion of Si-O-Al Peak Area in Si 2p Spectrum**
$Ni/Al_2O_3/SiC$	0.38	0.11
$Ni-16CeO_2/Al_2O_3/SiC$	0.52	0.31

Table S3 Performance comparison of representative Ni-based CO_2 methanation catalysts

Catalyst	Type	Temperature (°C)	WHSV ($mL \cdot g^{-1} \cdot h^{-1}$)	Catalyst mass (g)	CO_2 conversion (%)
$Ni/Al_2O_3/SiC$	Structured	400	36000	0.1	46.8
$Ni\text{-}16CeO_2/Al_2O_3/SiC$	Structured	400	36000	0.1	71.6
$Ni/CeO_2\text{-}ZrO_2/SiC$ foam	Structured	400	47027	0.05	71.1
$Ni/CeO_2/Al_2O_3$	Powder	400	30000	0.1	66.2

Catalyst	CH_4 selectivity (%)	Stability duration (h)	Activity loss (%)	Ref.
$Ni/Al_2O_3/SiC$	95	80	15.0	[21]
$Ni\text{-}16CeO_2/Al_2O_3/SiC$	95	80	7.8	-
$Ni/CeO_2\text{-}ZrO_2/SiC$ foam	94.2	100	8.5	[14]
$Ni/CeO_2/Al_2O_3$	97.2	60	9.3	[41]

Table S4 CO pulse chemisorption results, metal dispersion, and crystallite size of Ni-16CeO_2/Al_2O_3/SiC and Ni/Al_2O_3/SiC catalysts.

Catalyst	Cum. uptake (mmol/g)	Metal dispersion (%)	Crystallite size (hemisphere, nm)
Ni/Al_2O_3/SiC	4.03×10^{-4}	0.74	119.2
Ni-16CeO_2/Al_2O_3/SiC	4.63×10^{-4}	0.85	137.1
